# Supplementary material for: Evidence that a positive feedback loop drives centrosome maturation in fly embryos
Source: eLife. 2019 Sep 9;8:e50130. doi: 10.7554/eLife.50130 (PMC6733597; doi:10.7554/eLife.50130)

Cep215/Cdk5Rap2

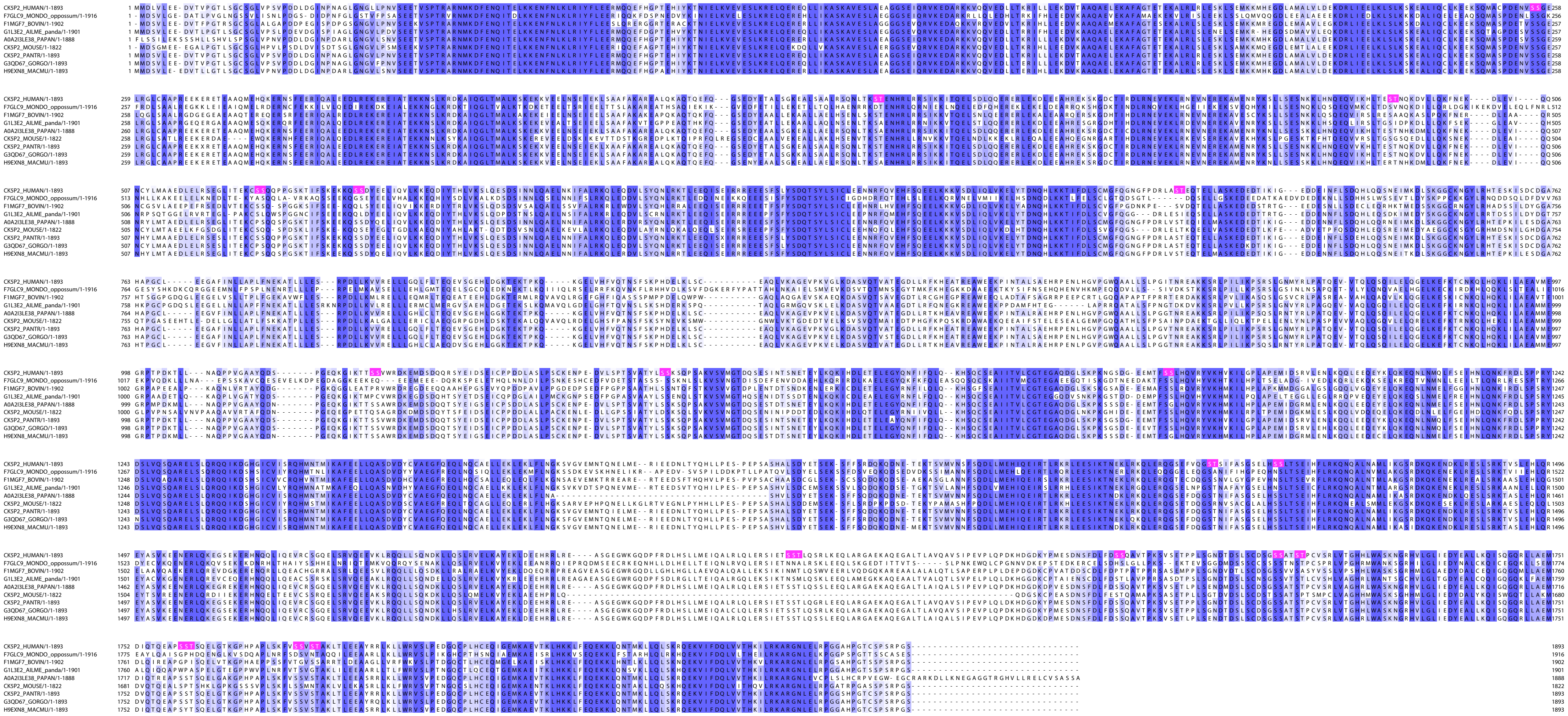

Supplement: Figure 2—figure supplement 1—source data 3. [file elife-50130-fig2-figsupp1-data3.pdf]
