# Supplementary material for: Evidence that a positive feedback loop drives centrosome maturation in fly embryos
Source: eLife. 2019 Sep 9;8:e50130. doi: 10.7554/eLife.50130 (PMC6733597; doi:10.7554/eLife.50130)
Supplement: Supplementary file 1. [file elife-50130-supp1.docx]

**Key Resources Table**

| **Reagent type (species) or resource** | **Designation** | **Source or reference** | **Identifiers** | **Additional information** |
| --- | --- | --- | --- | --- |
| Recombinant DNA reagent | pDONR-Zeo vector | Thermo Fisher |  |  |
| Recombinant DNA reagent | Ubq-GFPCT Gateway vector | (Basto et al., 2008) |  |  |
| Recombinant DNA reagent | Ubq-mCherryCT Gateway vector | (Basto et al., 2008) |  |  |
| Recombinant DNA reagent | pRNA-mKate2CT Gateway vector | (Novak et al., 2016) |  |  |
| Recombinant DNA reagent | pETM44 vector | EMBL |  |  |
| Recombinant DNA reagent | *spd-2* (pOT2 vector) | Geneservice Ltd | Clone LD24702 |  |
| Sequence-based reagent (primer) | Spd-2_AttB_FP_mutant | This paper |  | ggggacaagtttgtacaaaaaagcaggcttcacaagtttgtacaaaaaagcaggcttcatggacactaccagtggaagcc |
| Sequence-based reagent (primer) | Spd-2_AttB_FP_WT | This paper |  | ggggacaagtttgtacaaaaaagcaggcttcacaagtttgtacaaaaaagcaggcttcatggacagtagcagtggaagcc |
| Sequence-based reagent (primer) | Spd-2_AttB_RV | This paper |  | ggggaccactttgtacaagaaagctgggtcaaatttaaaactaatcgggacactgatgc |
| Sequence-based reagent (primer) | M1F  spd-2 S121A | This paper |  | ccaagggcacaaacatcgcttttgagcctgcggag |
| Sequence-based reagent (primer) | M1R rev  spd-2 S121A | This paper |  | ctccgcaggctcaaaagcgatgtttgtgcccttgg |
| Sequence-based reagent (primer) | M2F  spd-2 T329A | This paper |  | ccagaaagtaacgtggctctggattcggttggcgag |
| Sequence-based reagent (primer) | M2R rev  spd-2 T329A | This paper |  | ctcgccaaccgaatccagagccacgttactttctgg |
| Sequence-based reagent (primer) | M3F  spd-2 S397A | This paper |  | cgagatcctaagtctcgccgcgatcgacaaggcgc |
| Sequence-based reagent (primer) | M3R rev  spd-2 S397A | This paper |  | gcgccttgtcgatcgcggcgagacttaggatctcg |
| Sequence-based reagent (primer) | M4F  spd-2 S484A | This paper |  | cgcgcaagccgctcgctccgctggcggaccatc |
| Sequence-based reagent (primer) | M4R rev  spd-2 S484A | This paper |  | gatggtccgccagcggagcgagcggcttgcgcg |
| Sequence-based reagent (primer) | M5F  spd-2 S569A | This paper |  | gccagtgaacaagaagagggttgcgatcgctacaatggg |
| Sequence-based reagent (primer) | M5R rev  spd-2 S569A | This paper |  | cccattgtagcgatcgcaaccctcttcttgttcactggc |
| Sequence-based reagent (primer) | M6F  spd-2 S614A, S617,  S618A | This paper |  | ctggccaaaaatctagcgcccctggccgcgccaagaagctgtctc |
| Sequence-based reagent (primer) | M6R rev  spd-2 S614A, S617,  S618A | This paper |  | gagacagcttcttggcgcggccaggggcgctagatttttggccag |
| Sequence-based reagent (primer) | M7F  spd-2 T672A, S673A  and S674A | This paper |  | ggaagacgtgggttgggagccgccgctgttgcagtgccacgtag |
| Sequence-based reagent (primer) | M7R rev  spd-2 T672A, S673A  and S674A | This paper |  | ctacgtggcactgcaacagcggcggctcccaacccacgtcttcc |
| Sequence-based reagent (primer) | Spd2S625A_FW | This paper |  | cctgtcctcgccaagaagctgtctctcggcgccgctgctggacagcacaaccagttcc |
| Sequence-based reagent (primer) | Spd2S625A_RV | This paper |  | ggaactggttgtgctgtccagcagcggcgccgagagacagcttcttggcgaggacagg |
| Sequence-based reagent (primer) | Spd2T516A_FW | This paper |  | cagaagccgaggcggacatcgatgagtggccctcggcgccggtaaaggagcctagccgcagagtgagacg |
| Sequence-based reagent (primer) | Spd2T516A_RV | This paper |  | cgtctcactctgcggctaggctcctttaccggcgccgagggccactcatcgatgtccgcc |
| Sequence-based reagent (primer) | pRNA_PCR_FW | This paper |  | gacccagctttcttgtacaaagtg |
| Sequence-based reagent (primer) | pRNA_PCR_RV | This paper |  | gaagcctgcttttttgtacaaacttg |
| Sequence-based reagent (primer) | Spd2_Insert1m_FW | This paper |  | tgtacaaaaaagcaggcttcatggacactaccagtggaag |
| Sequence-based reagent (primer) | Spd2_Insert1146_RV | This study |  | tgtacaagaaagctgggtcaaatttaaaactaatcgggacactgatgc |
| Sequence-based reagent (primer) | Spd2_Insert1wt_FW | This paper |  | tgtacaaaaaagcaggcttcatggacagtagcagtggaag |
| Sequence-based reagent (primer) | Spd2_Insert552_FW | This paper |  | ctgcaccgaggatgaaaacgatgaggagga |
| Sequence-based reagent (primer) | Spd2_Insert552_RV | This paper |  | tcctcctcatcgttttcatcctcggtgcag |
| Sequence-based reagent (primer) | HiFi-DSpd-2(Mid)_FW | This paper |  | aggaaaacctgtacttccagggtactggctcgaatctcggacg |
| Sequence-based reagent (primer) | HiFi-DSpd-2(Mid)_RV | This paper |  | tcggatccggtacctcattacatggcttggagtgtgatcg |
| Sequence-based reagent (primer) | Primer to amplify pDONR with Sas-6 3’UTR overhang (forward) | This paper |  | gatacacagcgacccagctttcttgtacaaagtt |
| Sequence-based reagent (primer) | Primer to amplify pDONR with Sas-6 promoter overhang (reverse) | This paper |  | ggttggggacgaagcctgcttttttgtacaaagt |
| Sequence-based reagent (primer) | Primer to amplify 2 kb upstream of Sas-6 (forward) | This paper |  | agcaggcttcgtccccaaccaaccctagaccc |
| Sequence-based reagent (primer) | Primer to amplify 2 kb upstream of Sas-6 (reverse) | This paper |  | tgctcaccatgacagatcaggctcgtaagaaac |
| Sequence-based reagent (primer) | Primer to amplify NeonGreen with Sas-6 promoter overhang (forward) | This paper |  | ctgatctgtcatggtgagcaagggcgaggagg |
| Sequence-based reagent (primer) | Primer to amplify NeonGreen with Sas-6 3’UTR overhang (reverse) | This paper |  | ataagtaaaattacttgtacagctcgtccatgc |
| Sequence-based reagent (primer) | Primer to amplify 1 kb downstream of Sas-6 (forward) | This paper |  | gtacaagtaattttacttattgttaatgcattttttc |
| Sequence-based reagent (primer) | Primer to amplify 1 kb downstream of Sas-6 (reverse) | This paper |  | aagctgggtcgctgtgtatccatcttggccgc |
| Sequence-based reagent (primer) | Primer to introduce a second copy of NeonGreen with linker (forward) | This paper |  | gcggccgccacttgtacagctcgtccatgccc |
| Sequence-based reagent (primer) | Primer to introduce a second copy of NeonGreen with linker (forward) | This paper |  | gctgtacaagtggcggccgctcgagatggtga |
| Sequence-based reagent (primer) | Primer to amplify pDONR with Plk4 3’UTR overhang (forward) | This paper |  | gtattgtgcggacccagctttcttgtacaaagtt |
| Sequence-based reagent (primer) | Primer to amplify pDONR with Plk4 promoter overhang (reverse) | This paper |  | ggcggaagacgaagcctgcttttttgtacaaagt |
| Sequence-based reagent (primer) | Primer to amplify 2 kb upstream of Plk4 (forward) | This paper |  | agcaggcttcgtcttccgcccttctacttctgc |
| Sequence-based reagent (primer) | Primer to amplify 2 kb upstream of Plk4 (reverse) | This paper |  | tgctcaccatagctagccttttttctgtagactta |
| Sequence-based reagent (primer) | Primer to amplify NeonGreen with Plk4 promoter overhang (forward) | This paper |  | aaggctagctatggtgagcaagggcgaggagg |
| Sequence-based reagent (primer) | Primer to amplify NeonGreen with Plk4 3’UTR overhang (reverse) | This paper |  | tttgattcaattacttgtacagctcgtccatgc |
| Sequence-based reagent (primer) | Primer to amplify 1 kb downstream of Plk4 (forward) | This paper |  | gtacaagtaattgaatcaaaacttaattccaaaata |
| Sequence-based reagent (primer) | Primer to amplify 1 kb downstream of Plk4 (reverse) | This paper |  | aagctgggtccgcacaatacaacgaacagtgaac |
| Genetic reagent (*Drosophila melanogaster*) | Ubq-Spd-2-GFP | (Dix and Raff, 2007) | Spd-2-WT-GFP;  FLYB: FBtp0051369 |  |
| Genetic reagent (*D. melanogaster*) | Ubq-Spd-2^11A^-GFP | This paper | Spd-2-11A-GFP |  |
| Genetic reagent (*D. melanogaster*) | Ubq-Spd-2^CONS^-GFP | This paper | Spd-2-CONS-GFP |  |
| Genetic reagent (*D. melanogaster*) | Ubq-Spd-2^ALL^-GFP | This paper | Spd-2-ALL-GFP |  |
| Genetic reagent (*D. melanogaster*) | Ubq-Spd-2-mCherry | This paper | Spd-2-WT-mCherry |  |
| Genetic reagent (*D. melanogaster*) | Ubq-Spd-2^CONS^- mCherry | This paper | Spd-2-CONS-mCherry |  |
| Genetic reagent (*D. melanogaster*) | Ubq-Spd-2^ALL^- mCherry | This paper | Spd-2-ALL-mCherry |  |
| Genetic reagent (*D. melanogaster*) | Ubq-Jupiter-mCherry | (Callan et al., 2010) |  |  |
| Genetic reagent (*D. melanogaster*) | ePolo-GFP(Trap) | (Buszczak et al., 2006) | FLYB: FBal0211802;  RRID:BDSC_51552 |  |
| Genetic reagent (*D. melanogaster*) | Ubq-RFP-Cnn | (Conduit and Raff, 2010) | FLYB: FBtp0056991 |  |
| Genetic reagent (*D. melanogaster*) | eAsl-mKate2 | (Aydogan et al. 2019) |  |  |
| Genetic reagent (*D. melanogaster*) | p(Sas-6)-mNeonGreen | This study |  |  |
| Genetic reagent (*D. melanogaster*) | p(Sas-6)-dNeonGreen | This study |  |  |
| Genetic reagent (*D. melanogaster*) | p(Plk4)-mNeonGreen | This study |  |  |
| Genetic reagent (*D. melanogaster*) | Aur-A-GFP | (Lucas and Raff, 2007) |  |  |
| Genetic reagent (*D. melanogaster*) | *spd-2^z35711^* | (Giansanti et al., 2008) | FLYB: FBal0240470 |  |
| Genetic reagent (*D. melanogaster*) | *spd-2^G20143^* | (Dix and Raff, 2007) | FLYB: FBal0240486 |  |
| Genetic reagent (*D. melanogaster*) | *spd-2^Df(3L)st-j7^* | Bloomington Stock Center | Stock #5416;  FLYB: FBab0002416;  RRID:BDSC_5416 |  |
| Genetic reagent (*D. melanogaster*) | *cnn^f04547^* | (Lucas and Raff, 2007) | FLYB: FBal0180413; RRID:FlyBase_FBst1019233 |  |
| Antibody | Rabbit polyclonal anti-MBP | (Barros et al., 2005) | Lab ID: MBP | Animal#SK2860, initially raised against MBP-AurA; 1:500 |
| Antibody | Rabbit polyclonal anti-Spd-2 | (Dix and Raff, 2007) | Lab ID: Ab#57;  RRID:AB_2567456 | Animal#SK3428; 1:500 |
| Antibody | Rabbit polyclonal anti-Cnn | (Lucas and Raff, 2007) | Lab ID: Ab#37 | Animal#SK3516; 1:1000 |
| Antibody | Mouse monoclonal anti-γ-tubulin | Sigma | GTU-88; T6557; RRID:AB_477584 | 1:500 |
| Antibody | Mouse monoclonal anti-Actin | Sigma | A3853; RRID:AB_262137 | 1:500 |
| Antibody | Mouse monoclonal anti-GST | Thermo Fisher | MA4-004; RRID:AB_10979611 | 1:500 |
| Antibody | Rabbit polyclonal anti-GAGA factor | (Raff et al., 1994) | Lab ID: Ab#144 | Animal Z13-3; 1:500 |
| Antibody | Mouse monoclonal anti-α-tubulin | Sigma | DM1a; T9026; RRID:AB_477593 | 1:1000 |
| Antibody | Guinea pig polyclonal anti-Cnn | (Lucas and Raff, 2007) | Lab ID: Ab#55 | Animal#SK42; 1:1000 |
| Antibody | Rabbit polyclonal anti-Cnn pSer567 | (Feng et al., 2017) | Lab ID: Ab#236 | Animal#30129; 1:500 |
| Antibody | Sheep ECL anti-Mouse HRP-conjugated | GE Healthcare Life Sciences, Sigma | NA931V; RRID:AB_772193 |  |
| Antibody | Donkey ECL anti-Rabbit HRP-conjugated | GE Healthcare Life Sciences, Sigma | NA934V; RRID:AB_772191 |  |
| Antibody | Swine anti-Rabbit HRP conjugated | Dako, Agilent Technologies | P0399; RRID:AB_2728719 | 1:3000 |
| Antibody | Goat anti-Mouse IgG Alexa Flour 594 | Invitrogen, Thermo Fisher | A11032; RRID:AB_2534091 | 1:500 |
| Antibody | Llama GFP-Booster Atto488 | Chromotek | gba488; RRID:AB_2631386 | 1:500 |
| Antibody | Goat anti-Rabbit IgG Alexa Flour 594 | Invitrogen, Thermo Fisher | A11012; RRID:AB_2534079 | 1:500 |
| Antibody | Donkey anti-Guinea Pig IgG(H+L) CF405S | Biotium | 20356 | 1:500 |
| Chemical compound, drug | Alkaline phosphatase | Roche Diagnostics | 10713023 001 |  |
| Chemical compound, drug | Phosphatase inhibitor cocktails 2 and 3 | Sigma | P5726-1ML, P0044-1ML |  |
| Chemical compound, drug | Protein A Dynabeads (Life Technologies) | Thermo Fisher | 10002D |  |
| Chemical compound, drug | BS3 crosslinker | Thermo Fisher | 21580 |  |
| Chemical compound, drug | 10X Kinase buffer | CST | 9802 |  |
| Chemical compound, drug | ATP | CST | 9804 |  |
| Chemical compound, drug | SIGMAFAST EDTA-free protease inhibitor cocktail | Sigma | S883 |  |
| Peptide, recombinant protein | PLK1 | ProQinase | 0183-0000-1 |  |
| Peptide, recombinant protein | GST-Plk1-PBD | Sigma | SRP0360 |  |
| Commercial assay, kit | SuperSignal West Femto kit | Thermo Fisher | 34095 |  |
| Commercial assay, kit | NEBuilder® HiFi DNA Assembly Master Mix | New England Biolabs | E2621S |  |
| Commercial assay, kit | Gateway BP Clonase Enzyme mix | Thermo Fisher | 11789100 |  |
| Commercial assay, kit | Gateway LR Clonase Enzyme mix | Thermo Fisher | 11791100 |  |
| Commercial assay, kit | QuikChange Multi Site-Directed Mutagenesis kit | Agilent Technologies | 200515 |  |
| Commercial assay, kit | QuikChange II XL Site-Directed Mutagenesis kit | Agilent Technologies | 200521 |  |
| Commercial assay, kit | mMESSAGE mMACHINE T3 kit | Thermo Fisher | AM1348 |  |
| Commercial assay, kit | Dynabeads antibody coupling kit | Thermo Fisher | 14311D |  |
| Other | Vectashield without DAPI | Vector Laboratories | H-1000 |  |
| Other | Vectashield with DAPI | Vector Laboratories | H-1200 |  |
| Other | Beckman SW 41 Ti Rotor | Beckman Coulter |  |  |
| Other | LTQ Orbitrap Mass Spectrometer, UltiMate 3000 Nano LC system | Thermo Scientific, Advanced Proteomics Facility Oford |  |  |
| Other | Amylose resin | New England Biolabs | E8021L |  |
| Other | HiLoad 16/600 Superdex 200 column | GE Healthcare | 28989335 |  |
| Other | Perkin Elmer ERS Spinning Disk confocal system | PerkinElmer Inc. |  |  |
| Other | Zeiss Axioskop 2 microscope | Zeiss International |  |  |
| Other | DeltaVision OMX V3 Blaze microscope | GE Healthcare Life Sciences; Micron Oxford | 29065721 |  |
| Other | Zeiss 880 Airyscan microscope | Zeiss International; Micron Oxford |  |  |
| Software, algorithm | Jalview | (Waterhouse et al., 2009) | RRID:SCR_006459 | Version 2.10.4b1 |
| Software, algorithm | Mascot software | Matrix Science | RRID:SCR_014322 |  |
| Software, algorithm | ImageJ | NIH | RRID:SCR_003070 | Version 2.0.0 |
| Software, algorithm | Volocity | PerkinElmer Inc. | RRID:SCR_002668 | Version 6.3 |
| Software, algorithm | softWoRx | GE Healthcare Life Sciences |  | Version 6.1 |
| Software, algorithm | SIM-Check | (Ball et al., 2015) |  |  |
| Software, algorithm | OMX Editor | Micron Oxford |  | https://github.com/MicronOxford/OMXeditor |
| Software, algorithm | Chromagnon | (Matsuda et al., 2018) |  | https://github.com/macronucleus/Chromagnon |
| Software, algorithm | ZEN | Zeiss International | RRID:SCR_013672 | Black edition |
| Software, algorithm | Prism | GraphPad | RRID:SCR_002798 | Version 7 |
| Software, algorithm | R | R Core Team (2013) | RRID:SCR_001905 | http://www.R-project.org/ |
| Software, algorithm | OMERO | (Allan et al., 2012) | RRID:SCR_002629 | Version 5.4.5 |
| Software, algorithm | Illustrator/Photoshop | Adobe | RRID:SCR_010279; RRID:SCR_014199 | CS6 |

**Supplementary References**

Allan, C., Burel, J.-M., Moore, J., Blackburn, C., Linkert, M., Loynton, S., Macdonald, D., Moore, W.J., Neves, C., Patterson, A., et al. (2012). OMERO: flexible, model-driven data management for experimental biology. Nat Methods *9*, 245–253.

Aydogan, M.G., Wainman, A., Saurya, S., Steinacker, T.L., Caballe, A., Novak, Z.A., Baumbach, J., Muschalik, N., and Raff, J.W. (2018). A homeostatic clock sets daughter centriole size in flies. J Cell Biol *217*, 1233–1248.

Aydogan, M.G., Steinacker, T.L., Mofatteh, M., Gartenmann, L., Wainman, A., Saurya, S., Wong, S.S., Zhou, F.Y., Boemo, M.A., and Raff, J.W. (2019). An autonomous oscillator times and executes centriole biogenesis. BioRxiv 510875. Published online March 18, 2019. https://doi.org/10.1101/510875

Ball, G., Demmerle, J., Kaufmann, R., Davis, I., Dobbie, I.M., and Schermelleh, L. (2015). SIMcheck: a Toolbox for Successful Super-resolution Structured Illumination Microscopy. Sci Rep *5*, 15915.

Barros, T.P., Kinoshita, K., Hyman, A.A., and Raff, J.W. (2005). Aurora A activates D-TACC-Msps complexes exclusively at centrosomes to stabilize centrosomal microtubules. J Cell Biol *170*, 1039–1046.

Basto, R., Brunk, K., Vinadogrova, T., Peel, N., Franz, A., Khodjakov, A., and Raff, J.W. (2008). Centrosome amplification can initiate tumorigenesis in flies. Cell *133*, 1032–1042.

Bodenmiller, B., Malmstrom, J., Gerrits, B., Campbell, D., Lam, H., Schmidt, A., Rinner, O., Mueller, L.N., Shannon, P.T., Pedrioli, P.G., et al. (2007). PhosphoPep—a phosphoproteome resource for systems biology research in Drosophila Kc167 cells. Mol Syst Biol *3*.

Buszczak, M., Paterno, S., Lighthouse, D., Bachman, J., Planck, J., Owen, S., Skora, A.D., Nystul, T.G., Ohlstein, B., Allen, A., et al. (2006). The Carnegie Protein Trap Library: A Versatile Tool for Drosophila Developmental Studies. Genetics *175*, 1505–1531.

Callan, M.A., Cabernard, C., Heck, J., Luois, S., Doe, C.Q., and Zarnescu, D.C. (2010). Fragile X protein controls neural stem cell proliferation in the Drosophila brain. Human Molecular Genetics *19*, 3068–3079.

Conduit, P.T., and Raff, J.W. (2010). Cnn dynamics drive centrosome size asymmetry to ensure daughter centriole retention in Drosophila neuroblasts. *20*, 2187–2192.

Conduit, P.T., Feng, Z., Richens, J.H., Baumbach, J., Wainman, A., Bakshi, S.D., Dobbelaere, J., Johnson, S., Lea, S.M., and Raff, J.W. (2014a). The centrosome-specific phosphorylation of Cnn by Polo/Plk1 drives Cnn scaffold assembly and centrosome maturation. Dev Cell *28*, 659–669.

Conduit, P.T., Richens, J.H., Wainman, A., Holder, J., Vicente, C.C., Pratt, M.B., Dix, C.I., Novak, Z.A., Dobbie, I.M., Schermelleh, L., et al. (2014b). A molecular mechanism of mitotic centrosome assembly in Drosophila. Elife *3*, e03399.

Dix, C.I., and Raff, J.W. (2007). Drosophila Spd-2 recruits PCM to the sperm centriole, but is dispensable for centriole duplication. *17*, 1759–1764.

Feng, Z., Caballe, A., Wainman, A., Johnson, S., Haensele, A.F.M., Cottee, M.A., Conduit, P.T., Lea, S.M., and Raff, J.W. (2017). Structural Basis for Mitotic Centrosome Assembly in Flies. Cell *169*, 1078–1089.e13.

Giansanti, M.G., Bucciarelli, E., Bonaccorsi, S., and Gatti, M. (2008). Drosophila SPD-2 is an essential centriole component required for PCM recruitment and astral-microtubule nucleation. *18*, 303–309.

Habermann, K., Mirgorodskaya, E., Gobom, J., Lehmann, V., Müller, H., Blümlein, K., Deery, M.J., Czogiel, I., Erdmann, C., Ralser, M., et al. (2012). Functional Analysis of Centrosomal Kinase Substrates in Drosophila melanogaster Reveals a New Function of the Nuclear Envelope Component Otefin in Cell Cycle ….

Hu, Y., Sopko, R., Chung, V., Foos, M., Studer, R.A., Landry, S.D., Liu, D., Rabinow, L., Gnad, F., Beltrao, P., et al. (2018). iProteinDB: An Integrative Database of Drosophila Post-translational Modifications. G3 GenesGenomesGenetics *9*, 1–11.

Koenig, T., Menze, B.H., Kirchner, M., Monigatti, F., Parker, K.C., Patterson, T., Steen, J.J., Hamprecht, F.A., and Steen, H. (2008). Robust prediction of the MASCOT score for an improved quality assessment in mass spectrometric proteomics. J. Proteome Res. *7*, 3708–3717.

Lehmann, V., Müller, H., and Lange, B.M.H. (2006). Immunoisolation of centrosomes from Drosophila melanogaster. Curr Protoc Cell Biol *Chapter 3*, Unit3.17.

Lucas, E.P., and Raff, J.W. (2007). Maintaining the proper connection between the centrioles and the pericentriolar matrix requires Drosophila centrosomin. J Cell Biol *178*, 725–732.

Matsuda, A., Schermelleh, L., Hirano, Y., Haraguchi, T., and Hiraoka, Y. (2018). Accurate and fiducial-marker-free correction for three-dimensional chromatic shift in biological fluorescence microscopy. Sci Rep *8*, 7583.

Novak, Z.A., Conduit, P.T., Wainman, A., and Raff, J.W. (2014). Asterless licenses daughter centrioles to duplicate for the first time in Drosophila embryos. Curr. Biol. *24*, 1276–1282.

Novak, Z.A., Wainman, A., Gartenmann, L., and Raff, J.W. (2016). Cdk1 Phosphorylates Drosophila Sas-4 to Recruit Polo to Daughter Centrioles and Convert Them to Centrosomes. Dev Cell *37*, 545–557.

Raff, J.W., Kellum, R., and Alberts, B. (1994). The Drosophila GAGA transcription factor is associated with specific regions of heterochromatin throughout the cell cycle. Embo J *13*, 5977–5983.

Roberts, E.B. (1998). Drosophila A Practical Approach (Oxford University Press).

Tinevez, J.-Y., Perry, N., Schindelin, J., Hoopes, G.M., Reynolds, G.D., Laplantine, E., Bednarek, S.Y., Shorte, S.L., and Eliceiri, K.W. (2016). TrackMate: An open and extensible platform for single-particle tracking. Methods *115*, 80–90.

Waterhouse, A.M., Procter, J.B., Martin, D.M.A., Clamp, M., and Barton, G.J. (2009). Jalview Version 2--a multiple sequence alignment editor and analysis workbench. Bioinformatics *25*, 1189–1191.

Zhai, B., Villén, J., Beausoleil, S.A., Mintseris, J., and Gygi, S.P. (2008). Phosphoproteome analysis of Drosophila melanogaster embryos. J. Proteome Res. *7*, 1675–1682.
