# Supplementary material for: Evidence that a positive feedback loop drives centrosome maturation in fly embryos
Source: eLife. 2019 Sep 9;8:e50130. doi: 10.7554/eLife.50130 (PMC6733597; doi:10.7554/eLife.50130)
Supplement: Transparent reporting form [file elife-50130-transrepform.docx]

***eLife’s* transparent reporting form**

We encourage authors to provide detailed information *within their submission* to facilitate the interpretation and replication of experiments. Authors can upload supporting documentation to indicate the use of appropriate reporting guidelines for health-related research (see [EQUATOR Network](http://www.equator-network.org/%20)), life science research (see the [BioSharing Information Resource](https://biosharing.org/)), or the [ARRIVE guidelines](http://www.plosbiology.org/article/info:doi/10.1371/journal.pbio.1000412) for reporting work involving animal research. Where applicable, authors should refer to any relevant reporting standards documents in this form.

If you have any questions, please consult our Journal Policies and/or contact us: [editorial@elifesciences.org](mailto:editorial@elifesciences.org).

**Sample-size estimation**

- You should state whether an appropriate sample size was computed when the study was being designed
- You should state the statistical method of sample size computation and any required assumptions
- If no explicit power analysis was used, you should describe how you decided what sample (replicate) size (number) to use

Please outline where this information can be found within the submission (e.g., sections or figure legends), or explain why this information doesn’t apply to your submission:

No explicit power analysis was used, the sample size depends on embryo healthiness and how many embryos are laid per female in any particular experimental session.

Sample sizes are explained in every figure legend, further details are available in the materials and methods section (supplementary file).

**Replicates**

- You should report how often each experiment was performed
- You should include a definition of biological versus technical replication
- The data obtained should be provided and sufficient information should be provided to indicate the number of independent biological and/or technical replicates
- If you encountered any outliers, you should describe how these were handled
- Criteria for exclusion/inclusion of data should be clearly stated
- High-throughput sequence data should be uploaded before submission, with a private link for reviewers provided (these are available from both GEO and ArrayExpress)

Please outline where this information can be found within the submission (e.g., sections or figure legends), or explain why this information doesn’t apply to your submission:

Number of replicates and type of replication are indicated in the corresponding figure legends. The definition of biological versus technical replication is fully stated in the materials and methods.

Quantification data has been made available as source files.

No outliers encountered/removed.

Criteria for inclusion of data (for the 3D-SIM scoring analysis) has been detailed in the corresponding section of the materials and methods.

No high-throughput sequence data has been produced.

**Statistical reporting**

- Statistical analysis methods should be described and justified
- Raw data should be presented in figures whenever informative to do so (typically when N per group is less than 10)
- For each experiment, you should identify the statistical tests used, exact values of N, definitions of center, methods of multiple test correction, and dispersion and precision measures (e.g., mean, median, SD, SEM, confidence intervals; and, for the major substantive results, a measure of effect size (e.g., Pearson's r, Cohen's d)
- Report exact p-values wherever possible alongside the summary statistics and 95% confidence intervals. These should be reported for all key questions and not only when the p-value is less than 0.05.

Please outline where this information can be found within the submission (e.g., sections or figure legends), or explain why this information doesn’t apply to your submission:

Information on the details of the statistical analysis (test used, exact value of N, multiple test correction, mean and SD or SEM) can be found where applicable in the corresponding figure legends, as well as the materials and methods. No key questions rely on the results from statistical analysis so the exact p-values, summary statistics and confidence intervals have not been reported, but this can be amended if deemed necessary.

Because of the large N per group, examples of raw data are provided in the figures and all the raw data has been provided as source data files.

Measure of effect size is not applicable to this submission.

(For large datasets, or papers with a very large number of statistical tests, you may upload a single table file with tests, Ns, etc., with reference to sections in the manuscript.)

**Group allocation**

- Indicate how samples were allocated into experimental groups (in the case of clinical studies, please specify allocation to treatment method); if randomization was used, please also state if restricted randomization was applied
- Indicate if masking was used during group allocation, data collection and/or data analysis

Please outline where this information can be found within the submission (e.g., sections or figure legends), or explain why this information doesn’t apply to your submission:

This study uses *D. melanogaster* as its model organism, and flies (and the different tissue types) that were used to do all the experiments here were not processed through any specific selection or filtering process that would introduce bias into the experiments, therefore allowing random allocation.

Masking was used if the type of analysis required it, the use of masking is indicated in the corresponding figure legend and further details can be found in the materials and methods.

**Additional data files (“source data”)**

- We encourage you to upload relevant additional data files, such as numerical data that are represented as a graph in a figure, or as a summary table
- Where provided, these should be in the most useful format, and they can be uploaded as “Source data” files linked to a main figure or table
- Include model definition files including the full list of parameters used
- Include code used for data analysis (e.g., R, MatLab)
- Avoid stating that data files are “available upon request”

Please indicate the figures or tables for which source data files have been provided:

Source data files have been provided for Figure 1D and Figure 1-figure supplement 1D; Figure 2 and Figure 2-figure supplement 1; Figure3B,C; Figure 4; Figure 5; Figure 5-figure supplement 1; Figure 6; Figure 7C,D; Figure 7-figure supplement 1A,B and Figure 7-figure supplement 1E,F; Figure 8, Figure 8-figure supplement 1 and Figure 8-figure supplement 2; Figure 9B-E; Figure 9-figure supplement 1 and Figure 10F. Model details are stated in the materials and methods.

No specific software/code was developed specifically for data analysis for this study, all the tools used are publicly or commercially available and have been indicated in the materials and methods.
